# Cognitive control: modeling the impact on mental health

**DOI:** 10.3389/fpsyg.2025.1452714

**Published:** 2025-02-21

**Authors:** Leon Alker

**Affiliations:** Department of Psychology, Philipps University Marburg, Marburg, Germany

**Keywords:** cognitive control, mental health, affective neuroscience, computational modeling, clinical neuropsychology, mind control

## Abstract

The current study identified and investigated four leading models of the relationships among cognitive control, mental health, and psychological disorders. The Norwegian model of cognitive control emphasizes that the inability to disengage from irrelevant stimuli is related to a hyperreactive state of mind, high levels of anxiety, and deteriorated mental health. Motivational accounts of cognitive control highlight the decisive influence of perceived self-efficacy, which is positively related to mental health and negatively related to psychological disorders. Clinical-health psychological theories of cognitive control focus on the influence of cognitive control on the ability to regulate emotions. The dual competition model highlights the predominant impact of negative affect on cognitive control. This study had a cross sectional and descriptive design (*N* = 122). Ten Preacher and Hayes mediation analyses were conducted to compare all the models. The bootstrap sample was elevated to 5,000 to reach sufficient power for the statistical analyses. In sum, the findings of this study support most models. I propose a theoretical framework of cognitive control and mental health that integrates existing models and is applicable to various areas of life, such as clinical and neuropsychological practice, work, education, health, and personal relationships. This framework offers practical strategies for intervention and prevention, fostering resilience and well-being across various contexts while simultaneously reducing the risk of mental illnesses.

## 1 Introduction

### 1.1 Rationale

Cognitive control, the ability to rapidly adapt behavior in a changing world (Musslick and Cohen, 2021), has been identified as the most distinguishing characteristic of human behavior (Rosati, 2017) and is a key predictor of success in most areas of life (Miller et al., 2011; Diamond et al., 2007; Moffitt et al., 2011; Strömbäck et al., 2020). While this relation toward success is apparent, the relation between cognitive control, its antecedents and specific outcomes continues to be a task of complex and intricate matter. Olsen et al. (2018) noted that not cognitive control itself but rather its proactive and reactive subfunctions are significant in determining the consequences of disrupted cognitive control. Proactive control refers to the continuous anticipatory maintenance of goal-relevant information that recruits the lateral prefrontal cortex (PFC), while reactive control reflects a more transient, stimulus-driven goal reactivation mode that recruits the lateral PFC (plus a wider brain network) (Braver, 2012). Deficits in cognitive control have been associated with severe mental illnesses, e.g., schizophrenia, anxiety disorders, and depression (Lesh et al., 2011; Yu et al., 2018; Quinn et al., 2018), which is ‘the leading cause of disability around the world’ (Friedrich, 2017, p. 1). The role of cognitive control as a transdiagnostic risk factor for mental illnesses (McTeague et al., 2016) signifies the relevance of evaluating theories of cognitive control (the hyperreactive theory, the motivational theory, the clinical and health psychological theory of cognitive control and the dual competition framework), how it influences mental health and illness as two separate continua and factors that may be amenable to interventions (e.g., self-efficacy, distress tolerance, and emotional regulation).

## 2 Theories of cognitive control

The last two decades have been accompanied by a rapid growth of theories to explain inter-and intraindividual variabilities in cognition with respect to mental illness and positive mental health. Cognitive control, as a ‘paragon’ of higher cognition (Inzlicht et al., 2015), bridges the gap between neuroscience and clinical psychology in explaining the intricacies of mental illnesses. A variety of competing theories ranging from clinical-health psychology, motivational neuroscience, emotional research, and fMRI research emerged from the division of cognitive control into proactive and reactive cognitive control. Appreciating the division of cognitive control leads to testable predictions that can spur future research.

## 3 Dual-mechanism framework

The dual mechanisms framework (DMC) asserts that cognitive control operates via two distinct operating modes: ‘reactive control’ and ‘proactive control’ (Braver, 2012). Cognitive control is herein conceived as ‘the ability to regulate, coordinate, and sequence thoughts and actions in accordance with internally maintained behavioral goals’ (Braver, 2012, p.1). Proactive control refers to the early selection of goal-relevant information, which is actively maintained in the mind to prepare for cognitively demanding events and to bias perception, action systems and attention in a goal-driven manner (Braver, 2012; Miller and Cohen, 2001). Reactive control is merely activated after a high interference event is detected, with attention being mobilized as a ‘late correction’ mechanism (Braver, 2012; Jacoby et al., 1999). Successful cognition relies on a combination of both strategies, as both proactive and reactive cognitive control are complementary in nature and tend to be associated with advantages and limitations (Braver, 2012). Braver (2012) postulated that there is a bias insofar that individuals favor either a proactive or reactive control mode as a standard. This intraindividual variability in cognitive control may provide a framework for understanding changes in cognitive control in specific neuropsychiatric disorders. Thus, investigating both operating modes is a promising area for understanding inter-and intraindividual variabilities in cognition with respect to mental health.

## 4 Clinical and health psychological theory of cognitive control

Motivational and psychological deficits in cognitive control result from disparate mechanisms (Culbreth et al., 2018). Martins-Klein et al. (2020) suggested, proceeding from the DMC, that the distinction between antecedent (proactive strategies that engage sustained and preparatory cognitive control) and response-focused (reactive and transient) adaptive coping strategies is crucial for explaining the relation between cognitive control and mental illness. For example, individuals with cognitive control impairment and mental illness demonstrate preserved engagement of reactive control (and activity of the fronto-parietal network) but impaired recruitment of proactive cognitive control (and reduced parietal and DLPFC activity) (Lesh et al., 2013). That is, the enactment of reactive cognitive control appears to be preserved, while proactive cognitive control breaks down. In the context of the literature, proactive cognitive control deficits represent a more robust link to underlying neuropathophysiology, especially to disorgansed symptomatology (Lesh et al., 2013). These findings align with the emerging clinical perspective that cognitive control deficits are the underlying mechanism of maladaptive versus adaptive coping abilities in individuals with mental illness (Larrazabal et al., 2022). That is, the same brain regions responsible for greater persistence on behavioral DT tasks have been implicated in cognitive control (i.e., the right insula, bilateral medial frontal gyrus (MFG), anterior cingulate cortex (ACC), right ventromedial prefrontal cortex (vmPFC) and enhanced functional connectivity between the vmPFC subgenual ACC cluster and the right MFG) (Aron et al., 2003; Boes et al., 2008; Daughters et al., 2017; Japee et al., 2015; Menon and Uddin, 2010; Niendam et al., 2012; Shackman et al., 2011).

### 4.1 Distress tolerance and mental illness

Dysregulated behavior and low distress tolerance (DT) are common in individuals with cognitive control impairments (Gabrys et al., 2018; Macatee et al., 2018). DT refers to “the ability to effectively withstand aversive internal experiences, such as negative affect, traumatic experiences, and intrusive thoughts” (Larrazabal et al., 2022). Although DT on the surface seems to be similar to resilience, there is an important difference: DT is more amenable to interventions aimed at improving cognitive control and mental illness reduction because it is not a stable personality characteristic (Oshio et al., 2018). DT is assumed to affect the perception of stress and the evaluation of the consequences of experiencing negative emotional states, and individuals with low DT are overly prone and reactive to stress and distress (Riccardi et al., 2010). Consequently, they engage in maladaptive coping strategies to avoid inconvenient situations associated with negative emotional states. That is, individuals unable to withstand negative emotional states may not learn to become habituated to negative sensations and miss the opportunity to develop self-efficacy to manage those situations (Leyro et al., 2010). Inconvenient situations associated with negative emotional states can be avoided by using ineffective strategies such as avoidance, safety aids, and ritualising behaviors, which not only maintain but also potentiate those problems (Riccardi et al., 2010). DT has thus been associated with an array of mental illnesses, such as borderline personality disorder, substance abuse, self-injurious behavior, gambling (Anestis et al., 2007; Buckner et al., 2007; Daughters et al., 2005; Daughters et al., 2008; Gratz et al., 2006; Nock and Mendes, 2008), and anxiety-related problems, e.g., panic disorder, anxiety sensitivity, agoraphobia (Daughters et al., 2009; Marshall et al., 2008; Telch et al., 2003), PTSD (Fetzner et al., 2014), and suicidal behavior (Martin et al., 2018).

### 4.2 Distress tolerance, emotional regulation, and mental health

High DT appears to be of paramount importance to mental health, as it promotes adaptive emotional responses to life stressors (Larrazabal et al., 2022). In emotion research, DT is assumed to act as an emotion regulation (ER) ability that enables the selection and successful execution of specific ER strategies during stressful episodes (Larrazabal et al., 2022; Gainey et al., 2017; Tull and Aldao, 2015). In other words, DT is a more general individual difference factor that facilitates specific ER behaviors (Larrazabal et al., 2022). Consistently, high DT has been associated with emotional awareness, impulse control (Van Eck et al., 2017; Vujanovic et al., 2010) and mental health (Aldao et al., 2010; Jamieson et al., 2022). Specifically, individuals with high DT exhibit positive mental health outcomes and fewer mental health complaints (Aldao et al., 2010; Jamieson et al., 2022) because they possess ER strategies to alleviate potential vulnerabilities to mental health complaints (Larrazabal et al., 2022). Joint factor analyses of DT and ER with other constructs, such as mindfulness, negative urgency, anxiety sensitivity, intolerance of uncertainty, and experiential avoidance, suggest that they can be viewed as manifestations of a single latent dimension capturing adaptive coping abilities (Larrazabal et al., 2022; McHugh and Otto, 2012). Larrazabal et al. (2022) suggested that taxonomies of ER behaviors distinguish between maladaptive and adaptive strategies based on the direction and size of their empirical correlations with mental illnesses. In summary, high DT seems to foster healthy ER behaviors (e.g., affect labeling or problem solving) during stressful events, while low DT leads to counterproductive coping mechanisms (e.g., behavioral avoidance or suppression) (Jeffries et al., 2016; Larrazabal et al., 2022; Leyro et al., 2010).

## 5 Dual competition framework

In contrast to the aforementioned theory, the dual competition framework (DCF) purports that cognitive control modes are governed primarily by stimuli with emotional valence (Straub et al., 2020). The basic premise of the DCF is that motivation and emotion shape human cognition and behavior in an interactive manner (Pessoa, 2009). This framework is influential, as it (1) highlights the assumption of Braver (2012) that affect-related traits and states (i.e., anxiety, see Braver et al., 2009) may impact the preference of one cognitive control mode over another and (2) contrasts with the Clinical Psychological Theory of Cognitive Control’s proposition that the preference for cognitive control itself is responsible for ER strategies, the development of mental illnesses and positive mental health outcomes. Nevertheless, in line with the Clinical Theory of Cognitive Control, the DCF stresses that both control modes operate on different time scales (Straub et al., 2020). Studies using the DMC to advance this view are rare and primarily focus on anxiety, one of the strongest human emotions (Steimer, 2022). Anxiety is an aversive emotional and motivational state that occurs during and in anticipation of a threat (Eysenck et al., 2007). However, while Sarason (1988) found that anxiety impairs cognitive performance by increasing the allocation of attention resources to threat-related stimuli (internally and externally), Blankstein et al. (1990), Blankstein et al. (1989) showed that anxiety does not necessarily alleviate performance. This controversy culminated in the attention control theory (ACT), developed by Eysenck et al. (2007), to reconcile both findings.

### 5.1 Attentional control theory and negative affect

The ACT proposed that although negative affect (NA) influences processing efficiency, compensatory processes intervene in spare performance (Eysenck et al., 2007). According to the ACT, NA impairs processing efficiency by restricting the capacity of working memory, a finding that has been supported by several studies (Darke, 1988; Stout and Rokke, 2010; Moran, 2016). Proactive control relies on goal-directed attentional control, which is dependent on working memory (Duncan et al., 1996; Kane and Engle, 2002; Braver, 2012); thus, NA was posited to impair proactive control (Moser et al., 2016), inhibition (Eysenck et al., 2007; White et al., 2011) and attentional control (Coombes et al., 2009). These reduced capacities, in turn, require individuals to rely more on reactive control during stimulus-driven attention (Yang et al., 2018). Correspondingly, fMRI studies revealed that NA induction led to a shift from sustained to transient activation in regions activated in working memory (WM) regions (Fales et al., 2008). Yang et al. (2018) argued that NA is associated with alleviated proactive control and enhanced reactive control because sustained activity subserves proactive control and transient activity reactive control. Recently, Valadez et al. (2021) reported that this pattern led to behavioral inhibition, which can cause mental illnesses (Hirshfeld-Becker et al., 2008). This view is best summarized by Inzlicht et al. (2015): “Often seen as the paragon of higher cognition, here we suggest that cognitive control is dependent on emotion. Rather than asking whether control is influenced by emotion, we ask whether control itself can be understood as an emotional process […]. Critically, we propose that emotion is not an inert byproduct of conflict but is instrumental in recruiting control.” Nevertheless, following theory illustrates what happens if reactive cognitive control is overly employed.

## 6 Theory of hyperreactive cognitive control

Dysregulated, hyperreactive cognitive control poses a critical risk to mental health and has been associated with difficulties in everyday life. Olsen et al. (2018) showed that white matter connectivity between fronto-parietal brain regions is central to realizing proactive control and fluid intelligence. Conversely, hyperreactive cognitive control processing has been linked to anxiety (Burgess and Braver, 2010; Fales et al., 2008), impoverished white matter organization (Olsen et al., 2018), and decreased fluid intelligence (Burgess and Braver, 2010). Olsen et al. (2018) suggested that the ‘behavioural phenotype’ of those with impoverished white matter connectivity primarily exhibits difficulties with a high demand for proactive control while exhibiting hyperreactive cognitive control. This altered hemodynamics is assumed to underlie common difficulties in everyday life, as a well-regulated balance between being able to quickly react to sudden stimuli and proactive planning behavior is crucial for adaptive functioning (Olsen et al., 2018). Burgess and Braver (2010) reported that during interference tasks, cognitively healthy individuals rely more on reactive cognitive control processing during low expectancy conditions and more on proactive cognitive control processing during high expectancy conditions. In other words, the suboptimal organization of the central nervous system leads to a hyperreactive brain state of increased vigilance to low-frequency events (Olsen et al., 2018). That is, hyperreactive cognitive control leads to sensory overload and sensory processing problems (i.e., sensory modulation and integration; see Koziol et al., 2011) by disrupting mechanisms of selective attention, which are necessary to focus on relevant stimuli and events while shielding us from irrelevant stimuli (Jensen and Weng, 2019).

### 6.1 Theory of sensory modulation

Theories of sensory modulation refer to the process by which the brain regulates itself (Ayres, 1979) insofar as the central nervous system (CNS) changes the excitability and responsiveness of neuronal circuits to adjust to external conditions (Noback et al., 1996). This process relies on excitatory sensitization and habituation (Gere et al., 2009). Habituation emerges when the CNS recognizes a stimulus as repetitive or familiar and leads to neural inhibition (Dunn, 1997). Without habituation, an individual is continuously distracted by new stimuli (Gere et al., 2009). Olsen et al. (2018) suggested that the poor white matter organization of those who rely more on reactive cognitive control processing can result in too much attention being paid to irrelevant stimuli. Consequently, the ability to mediate incoming stimuli to focus on specific tasks while attending to one’s surroundings might thus be disturbed. Specifically, excitatory sensitization, which, through the process of sensory modulation, enhances attention and the immediate response to a stimulus by transducing stimuli from the environment into neural signals, might be beyond the acceptable range to facilitate an adaptive behavioral response (Dunn, 1997; Gere et al., 2009). Modulation may refer to physiological or behavioral adjustments as responses to sensory stimulation (McIntosh et al., 1999). This process can be unstable in those who rely more on reactive cognitive processing because their neurological threshold for irrelevant stimuli is too low.

### 6.2 Theory of sensory integration

Theories of sensory integration refer to the interactive relationship between physiological, behavioral, and emotional responses and neurological thresholds as a consequence of atypical modulation abilities (Gere et al., 2009; McIntosh et al., 1999). Sensory modulation is necessary for the body and brain to maintain homeostasis by modulating new and ongoing stimuli (Gere et al., 2009), and modulation inability has been associated with psychophysiological disruptions of sympathetic and parasympathetic reactions (McIntosh et al., 1999; Schaaf et al., 2003). Even typical and benign sensory stimuli may thus be experienced as stressful (Wilbarger and Wilbarger, 2022), unpleasant (Bar-Shalita et al., 2008), irritating (Kinnealey et al., 1995), or painful (Roley et al., 2001), rendering the individual unable to appropriately respond to stimuli and to react instead with withdrawal or defensive behaviors (Bar-Shalita et al., 2008). Furthermore, Gere et al. (2009) suggested that sensory processing problems negatively affect emotional, social and psychological well-being, which can thus deteriorate overall mental health (according to Westerhof and Keyes, 2010, mental health is composed of social, emotional and psychological well-being). Similarly, levels of mental illness may increase because sensory processing problems disturb physiological homeostasis (Feder et al., 2010) and reduce resilience (Vötter, 2019). This, in sum, deteriorates the mental health (Alker and Radstaak, 2024).

## 7 Motivational theory of cognitive control

Exerting cognitive control comes at a cost. We experience the exertion of cognitive control as effortful, which therefore needs some sort of incentive or justification (Frömer et al., 2021). Research has shown that individuals are more inclined to exert mental effort on a cognitive control task when they are offered a greater reward if they perform well (Croxson et al., 2009; Dixon and Christoff, 2012; Padmala and Pessoa, 2011; Parro et al., 2018; Hall-McMaster et al., 2019; Kool and Botvinick, 2014; Krebs et al., 2012; Schmidt et al., 2012; Vanessa et al., 2014; Yee et al., 2016). However, the expectation may not translate into behavior if the perceived self-efficacy to implement the respective behavior is low, even if much is at risk (Frömer et al., 2021). According to this model, individuals integrate the self-efficacy of task performance and the expected reward to determine the expected value of control and subsequently adjust their allocated control (Frömer et al., 2021). The expected value of control (EVC) of a given control allocation is determined by weighing the benefits against the costs that need to be incurred (mental effort) by the task (Frömer et al., 2021). The benefits are a function of both the expected outcomes for reaching one’s aim (e.g., becoming healthy) and the likelihood of reaching that aim with a certain investment of control (e.g., becoming healthy by actively participating in treatment activities). In sum, motivational accounts of cognitive control highlight the significance of mental effort, effort costs, and self-efficacy in explaining the impact of cognitive control on mental health (Grahek et al., 2019).

### 7.1 Cognitive control, mental effort, and mental health

Impaired motivation is a key feature of mental illnesses, disturbed functioning, mental health (Culbreth et al., 2018) and the allocation of cognitive control (Grahek et al., 2019). The allocation of cognitive control is driven by goals (Grahek et al., 2019). While hopelessness and an external locus of control are related to an array of mental illness (Gan et al., 2022; Maier and Seligman, 2016; Seligman, 1972) and have also been associated with reduced willingness to exert mental effort (Marchetti et al., 2018), the opposite applies at the prospect of reward, which enhances cognitive control processes (Botvinick and Braver, 2015; Krebs et al., 2012). The allocation of cognitive control thus relies on outcome valance and controllability, which, in turn, are related to self-efficacy levels (Grahek et al., 2019). Resultingly, individuals with mental illnesses are less likely or willing to exert more effort, for example, for proactive cognitive control, and rely more on automatic cognitive control, such as reactive cognitive control. However, sufficient effort needs to be expended to override automatic processes to reach a goal or to break detrimental and ineffective coping strategies often prevalent in mental illnesses, such as safety aids, ritualising behaviors, and avoidance. Individuals who receive the opportunity to develop self-efficacy to manage inconvenient situations develop a greater sense of self-mastery and DT (Leyro et al., 2010). Consequently, the willingness to exert mental effort enhances the probability of changing existing low self-efficacy, controllability estimates and beliefs (Grahek et al., 2019). In sum, self-efficacy (i.e., controllability estimates and beliefs) is crucial for exerting mental effort and allocating cognitive control.

### 7.2 Motivation, controllability, and locus of control

Motivational accounts are central to controllability estimates, the locus of control, and self-efficacy (Culbreth et al., 2018). Recent experimental work has suggested that individuals with mental illness are less willing to exert effort to obtain a reward or outcome because they exhibit motivational impairment (Culbreth et al., 2018). Motivation relies on outcome controllability, outcome value and self-efficacy. Outcome controllability refers to the extent to which individuals believe that contrary to external forces (beyond their control), they have control over the outcome of events in their lives (Galvin et al., 2018). Locus of control is crucial for an individual’s security, well-being and mental health, which has been supported by several lines of research (Leotti et al., 2010). An external locus of control that is repetitively and habitually endorsed leads to learned helplessness, low self-efficacy, and anhedonia, one of the leading symptoms of depression (Pizzagalli, 2014; Prihadi et al., 2018). However, even if the locus of control is internally attributed, the expectation may not translate into behavior if the perceived self-efficacy of the respective behavior and thus the likelihood of achieving the desired aim is low (Frömer et al., 2021). Thus, self-efficacy levels will determine whether goal-directed and proactive behavior is performed since the expectation may not translate into behavior if self-efficacy is low, even if the stakes (i.e., the outcome value) of not implementing the behavior are high (Frömer et al., 2021). Abramson et al. (1978), Maier and Seligman (2016) and Seligman (1972) demonstrated that learned helplessness and self-efficacy are of paramount importance for understanding mental health and mental illnesses, e.g., depression and anxiety.

### 7.3 Perceived self-efficacy

The ubiquitous and pervasive relevance of self-efficacy for emotional and behavioral functioning was already recognized during the 1990s (Maddux, 1995). Since then, it has become one of the top tier constructs in psychology, medicine, sociology, nursing, and other related fields (Maddux and Gosselin, 2012). Perceived self-efficacy, hereafter referred to as self-efficacy, refers to “the person’s confidence in his or her ability to perform a behaviour” (LaMorte, 2016). Self-efficacy as a construct has evolved to explain why some individuals fail or are unwilling to engage in behaviors that are clearly within their repertoire. Bandura (1983) stressed that there is a marked discrepancy between possessing skills and being able to make use of them in diverse circumstances. Consequently, different individuals with the same skills may adjust and adapt poorly, adequately, or exceptionally to life’s infinite challenges. Widmer et al. (2014) suggested that self-efficacy is even more relevant than an individual’s actual abilities. Despite the presumed explanatory power of this concept, little research on perceived self-efficacy is available in clinical neuropsychology, although preliminary evidence in neuroscience seems to support the social-psychological tenets of self-efficacy at multiple levels (for a review, see Stone, 2018). Although the relationship between self-efficacy and cognitive control has been theoretically established (Frömer et al., 2021), it is not clear whether this effect is bidirectional or reversed. Siegle et al. (2007), for example, assume that cognitive control itself enhances self-efficacy. This research aims as a secondary goal to clarify the relationships among self-efficacy, DT, mental health and illness by means of applied experiments in the field of neuropsychology.

### 7.4 Bootstrapping mediation analysis

Bootstrapping is a robust non-parametric statistical method that allows researchers to estimate the sampling distribution of a statistic by resampling with replacement from the original dataset. This technique is valuable when the theoretical distribution of the statistic is unknown or difficult to derive (Efron and Tibshirani, 1993). By generating multiple resampled datasets, researchers can construct empirical distributions of the statistic, which can be used to derive confidence intervals, perform hypothesis testing, and assess the precision of estimates (Davison and Hinkley, 1997). This approach is different from traditional parametric methods that often require a power analysis to determine the sample size needed to detect an effect with a certain level of confidence (Preacher and Hayes, 2014). In bootstrap mediation analysis, the data is resampled multiple times (typically thousands of times) to create an empirical distribution of the indirect effect. This allows for the estimation of confidence intervals without relying on assumptions about the distribution of the data. As a result, the need for *a priori* power analysis is mitigated because the bootstrap method provides a direct assessment of the variability and significance of the indirect effect based on the observed data (Preacher and Hayes, 2014).

The bootstrap method involves following steps: (1) repeatedly drawing random samples (with replacement) from the original dataset, (2) calculating the statistic of interest for each resample, and (3) using the distribution of these resampled statistics to make inferences about the population parameter (Cogneau and Zakamouline, 2010). This approach is advantageous in addressing data with atypical distributions that deviate from parametric assumptions, providing a more reliable alternative to traditional parametric methods (Kostanek et al., 2024). The Preacher and Hayes (2024) bootstrap method circumvents limitations of traditional approaches as it assesses the most likely directions by interchanging the dependent and independent variables by comparing the strongest impact sizes of the respective reversed models.

## 8 Present research

Based on the literature review, four leading strings of the relation between cognitive control, mental health and psychological disorders were identified. Motivational accounts of cognitive control suggest that perceived self-efficacy plays a predominant role in determining the impact on mental health and the development of psychological disorders. The Norwegian model of cognitive control emphasizes that the inability to disengage from irrelevant stimuli is related to a hyperreactive state of mind and deteriorated mental health. However, clinical-health psychological theories of cognitive control focus on the influence of cognitive control on the ability to regulate emotions. The dual competition framework highlights the ubiquitous impact of negative impacts on cognitive control.

### 8.1 Participants

This study was carried out in accordance with relevant guidelines and regulations and was approved by the ethics committee of Philipps-University Marburg, Germany. Informed consent was obtained from all 122 participants. Ninety-five participants were students, 27 of whom were employees of a university hospital. Students were recruited via the SONA first-year practicum and were then forwarded to the online survey platform SoSci Survey for study participation. SONA is an online participant management system used to recruit and schedule student participants, typically in exchange for course credit. SoSci Survey is an online survey platform used to administer questionnaires and collect data.

### 8.2 Materials

#### 8.2.1 MHC-SF

The Mental Health Continuum-Short Form (MHC-SF) is a 14-item self-administered questionnaire used to measure social, psychological, and emotional well-being. It exhibits high internal consistency (Cronbach’s alpha = 0.89) and moderate test–retest reliability (*r* = 0.68) (Lamers et al., 2011). The three-factorial structure of the MHC-SF has been confirmed, with the three subscales correlating well with the respective aspects of well-being and functioning, establishing convergent validity (Lamers et al., 2011). Mental health can be differentiated from mental illness, showing discriminant validity (Lamers et al., 2011). On a 6-point Likert scale (1 = never to 6 = every day), participants rated how much they agreed with statements such as “That our society is becoming a better place for people?” Context and Limitations: The MHC-SF has been widely used in various populations, including adolescents and adults, to assess overall mental well-being. However, it is important to note that the self-report nature of the questionnaire may introduce response biases, and cultural differences may affect the interpretation of the results. The MHC-SF also included indices of social support and psychosocial variables, which is measured by social well-being.

#### 8.2.2 BSI-18

The Brief Symptom Inventory (BSI-18) is an 18-item self-report questionnaire used to identify clinically relevant psychological symptoms in adolescents and adults (Derogatis, 1975). The alpha coefficients for the BSI symptom scales exhibited satisfactory degrees of internal consistency (Cronbach’s alpha = 0.74 to 0.89) (Geisheim et al., 2002). Convergent validity was established by intercorrelations with clinical rating scales. On a 5-point Likert scale (1 = not at all to 5 = extremely), participants rate the extent to which they agree with statements such as “Feeling that you are watched or talked about by others.” Comparison with Other Instruments: The BSI-18 is often compared to other symptom inventories, such as the Symptom Checklist-90 (SCL-90), due to its brevity and ease of administration. Its shorter length makes it more practical for use in clinical settings where time is limited.

#### 8.2.3 SV-12

The Processing Problem Scale of the SV-12 is a 14-item self-administered questionnaire measuring sensory processing problems (Hinterberger et al., 2019). Cronbach’s alpha ranged from 0.77 to 0.84, indicating good internal consistency. Convergent validity was established by a two-factorial solution of the two separate scales of the SV-12 (Hinterberger et al., 2019). An example of a processing problem scale item is “I often feel that I need more time to process certain impressions or experiences.” Participants could rate the extent of their agreement on a 5-point Likert scale (1 = does not apply to 5 = applies completely).

#### 8.2.4 DTS

The Distress Tolerance Scale (DTS) is a 15-item self-administered questionnaire that measures emotional distress tolerance (Alker, 2024). The criterion for construct and convergent validity has been established, and test–retest reliability (*r* = 0.61) and internal consistency (Cronbach’s alpha = 0.82) were deemed good to excellent (Simon and Gaher, 2005). On a 5-point Likert scale (1 = strongly agree to 5 = strongly disagree), participants indicate the degree to which they feel distressed, e.g., “Feeling distressed or upset is unbearable to me.”

#### 8.2.5 PANAS

The Positive and Negative Affect Schedule (PANAS) is a 20-item self-administered questionnaire measuring positive and negative affect. In this study, only the negative affect scale was included. The negative affect scale exhibits good to excellent psychometric properties (Cronbach’s alpha = 0.85) (Crawford and Henry, 2004; Heubeck and Wilkinson, 2019). On a 5-point Likert scale (1 = strongly disagree to 5 = strongly agree), participants are asked to indicate how much they felt, e.g., during the last week, nervous or anxious.

#### 8.2.6 CCFQ

The Cognitive Control and Flexibility Questionnaire (CCFQ) is an 18-item self-administered questionnaire measuring cognitive control and emotional regulation. Construct, convergent, and incremental validity, as well as high reliability (Cronbach’s alpha = 0.88), have been established (Gabrys et al., 2018). The CCFQ can tap into a multitude of ways through which cognitive flexibility can be expressed in a single brief questionnaire. An example of a CCFQ item is “I control my thoughts and feelings by putting the situation into context.”

#### 8.2.7 SWE

The General Self-Efficacy Scale (SWE) is a 10-item self-administered questionnaire measuring a general sense of perceived self-efficacy (Free University of Berlin, 2023). Cronbach’s alphas ranged from 0.76 to 0.90 in samples from 23 nations, with the majority in the high 80s. The scale was found to be unidimensional. Criterion validity has been established internationally by numerous studies. On a 4-point Likert scale (1 = not at all true to 4 = exactly true), participants indicate the extent to which they agree with statements such as “I can always manage to solve difficult problems if I try hard enough.”

#### 8.2.8 HOME-21

The HOME-21 is a revised form of the HOME-Short Form. The HOME-21 measures the extent to which adults had a favorable childhood. The HOME-21 has been adapted for the purpose of this study as a self-report questionnaire, incorporating 28 items measuring the extent to which participants agree, on a 1-5-point Likert scale (1 = never to 5 = more than once a day), to statements such as “How often did you eat together with your family members” when they were a child. The HOME-21 exhibits good to excellent psychometric properties (Cronbach’s alpha = 0.83) (Lansford et al., 2023).

### 8.3 Procedure

The data collection period encompassed 99 days. It started on the 12^th^ of July 2024 and ended on the 18^th^ of October 2024. The questionnaires were transformed into an online questionnaire by using the survey website Sosci. To contact participants from the university hospital, an e-mail was forwarded to all employees. E-mail incorporated a link to the survey website Sosci. The link to the survey was published in Sona-Systems to contact university students. The Sona System is a platform where researchers can gather convenience-based samples consisting mainly of first-year students in need of European Credit Transfer System (ECTS) points. All participants were transferred to the website by clicking on the provided link. During the first part of the questionnaire, participants were informed about the topic of the study and were asked for their informed consent. Afterwards, the participants completed the eight surveys. After the completion of all the questionnaires, the participants were asked if they wanted to be informed about the results of this study and were offered an option to complete their e-mail address. Finally, they were thanked for their participation and were informed about the opportunity to contact the researchers.

The participants were university students and employees from a university hospital. The survey was programmed and implemented via Sosci. Child development was included as a potential confounder variable. For example, detrimental child development has been found to foster the endorsement of a more reactive – compared to a proactive – cognitive control style (Olsen et al., 2018).

A higher score on the “appraisal and coping flexibility” subscale of the CCFQ (Gabrys et al., 2018) was used as an indicator of proactive control, as a decrease in flexibility in cognitive control is regarded as the hallmark of reactive cognitive control (Mäki-Marttunen et al., 2019).

### 8.4 Design and analysis

This study had a descriptive and cross-sectional design. The data analysis was conducted with the statistical software SPSS. First, descriptive statistics were computed, including the means, standard deviations, kurtosis, and skewness of all variables. The Kolmogorov–Smirnov test was used to test for a normal distribution. Spearman or Pearson correlations were used if the data were normally distributed. The effect sizes of R2 were set at 0.01 (small), 0.09 (medium) and 0.25 (large) according to the recommendations of University of Cambridge (n.d.) and Cohen (1988) for determining the magnitude of the observed effects.

**Figure 1:**
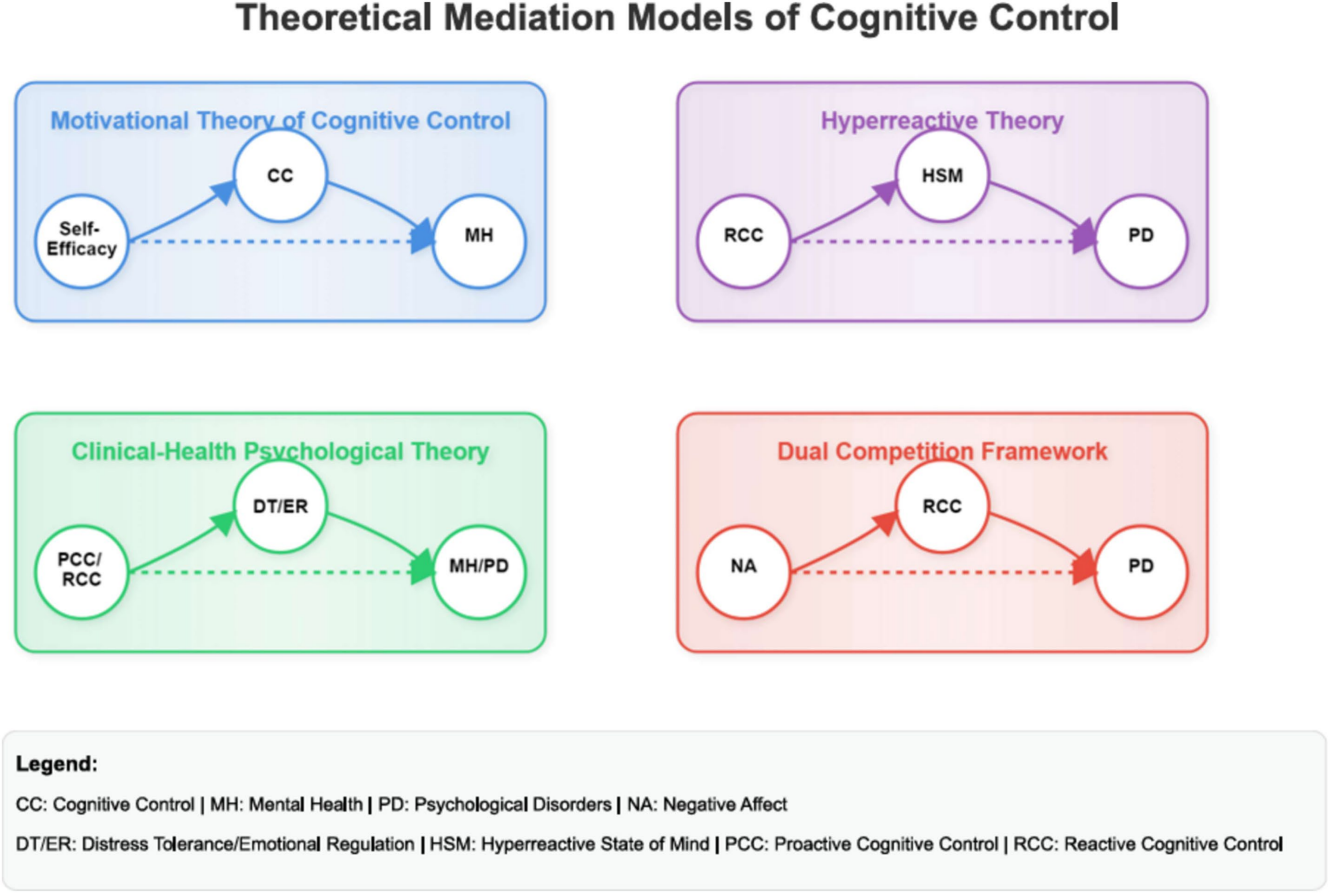
Conceptual models of the four theories of cognitive control, mental health and mental illness. PD refers to mental illness to emphasize the contrast to mental health as a positive resource-based continua. The concept of a hyperreactive state of mind was utilized to align with the constructs outlined by Olsen. Cognitive Control is utilized to delineate the distinct impacts of both proactive and reactive cognitive control. CC, cognitive control; MH, mental health; PD, psychological disorders; NA, negative affect; DT/ER, distress tolerance/emotional regulation; HSM, hyperreactive state of mind; PCC, proactive cognitive control; RCC, reactive cognitive control.

The ten mediation models (see Figure 1) were evaluated by conducting four mediational analyses, which determined the indirect effects of the mediator using the MEDIATE file developed by Hayes and Preacher (2014). Bootstrapping was chosen to test the mediation hypotheses because it is more effective than the Baron and Kenny (1986) approach and because it works with comparably low sample sizes by randomly selecting a large sample from the original sample, which increases the statistical power (Cerin et al., 2006). Unlike traditional assumptions of the Baron and Kenny method, the MEDIATE model allows for a more nuanced understanding of causal relationships (Preacher and Hayes, 2004; Rucker et al., 2011). The MEDIATE model does not assume the traditional assumptions of the Baron and Kenny (1986) causal steps approach, such as a significant relation between x and y (for an elaborate discussion: Preacher and Hayes, 2004; Rucker et al., 2011).

To check for reverse causal effects, *x* was interchanged with *m,* and effect sizes between these and the initial models were compared. It was assumed that effect sizes for the proposed models are substantially larger than those for the reverse causal effect models. The MEDIATE file provides these reverse causal effect models by default in the output. The reversed models are displayed first.

The indirect effects were computed to identify the mediating effects. The indirect effect was analyzed by calculating the non-standardized indirect effects for each of 5,000 bootstrapped samples. The corresponding 95% confidence intervals (CIs) were computed by determining the indirect effects at the 2.5^th^ and 95.5^th^ percentiles. An indirect effect of the mediation variable was deemed to be confirmed if the CI did not include the number zero (Preacher and Hayes, 2004). With a bootstrap sampling of 5,000, it is possible to circumvent power problems and to apply mediational analyses with more confidence.

## 9 Results

### 9.1 Descriptive analyses

The descriptive analyses and the zero-order correlations for all included variables are illustrated in Tables 1–3, respectively.

**Table 1:** The variables under study - St. Franziskus-Hospital.

Variable	*N*	Minimum	Maximum	Mean	Standard deviation
Self-Efficacy	27	1.27	3.27	2.64	0.39
Mental Illness	27	1.39	4.33	1.88	0.57
Distress Tolerance	27	1.40	5.00	3.69	0.98
Negative Affect	27	1.00	4.80	1.90	0.83
Emotional Regulation	27	1.22	6.11	4.32	1.11
Proactive & Reactive Cognitive Control	27	1.33	7.00	4.67	1.15
Sensory Processing Problems	27	1.57	4.43	3.17	0.77
Child Development	27	1.53	4.14	3.21	0.56

Descriptive analyses of the employees of the St. Franziskus-Hospital.

**Table 2:** The variables under study - Marburg University.

Variable	*N*	Minimum	Maximum	Mean	Standard Deviation
Self-Efficacy	95	0.91	3.55	2.52	0.45
Mental Illness	95	1.39	4.50	2.04	0.66
Distress Tolerance	95	1.60	5.00	3.45	0.84
Negative Affect	95	1.00	4.30	2.16	0.79
Emotional Regulation	95	2.11	6.11	3.99	0.87
Proactive & Reactive Cognitive Control	95	2.00	7.00	4.72	0.96
Sensory Processing Problems	95	1.43	4.29	2.90	0.72
Child Development	95	1.58	5.00	3.46	0.52

Descriptive analyses of students of the Philipps-University Marburg.

**Table 3:** Zero-order correlations for all in the study included variables.

	1.	2.	3.	4.	5.	6.	7.	8.
1. Mental Illness	1	-						
2. Distress Tolerance	−0.58**	1	-					
3. Negative Affect	0.81**	0.59**	1	-				
4. Emotional Regulation	−0.52**	0.64**	−0.62	1	-			
5. Proactive & Reactive Cognitive Control	−0.31**	0.25**	−0.26**	0.38**	1	-		
6. Sensory Processing Problems	−0.43**	0.43**	0.65**	−0.50**	0.62**	1	-	
7. Child Development	−0.29**	0.10	−0.23*	0.20*	0.07	0.13	1	-
8. Self-Efficacy	−46**	0.58**	−0.45**	0.54**	0.29**	0.60**	0.10	1

All variables, excepted child development, correlate highly with the other variables. *p < 0.05, **p < 0.01.

The proactive and reactive cognitive scores had a skewness of −0.39 (SE = 0.22) and a kurtosis of 0.57 (SE = 0.44). DT scores were normally distributed, with a skewness of −0.21 (SE = −0.21) and a kurtosis of −0.70 (SE = −0.44). The proactive and reactive cognitive control scores were normally distributed, with a skewness of −0.39 (SE = 0.22) and a kurtosis of 0.57 (SE = 0.44). DT scores were normally distributed, with a skewness of −0.21 (SE = −0.21) and a kurtosis of −0.70 (SE = −0.44). The ER scores were normally distributed, with a skewness of −0.17 (SE = 0.22) and a kurtosis of 0.03 (SE = 0.44). The scores for the sensory processing problems variable were normally distributed, with a skewness of −0.17 (SE = 0.22) and (0.22) and a kurtosis of 1.27 (SE = 0.44). The scores on the child development scale were nonnormally distributed, with a skewness of −0.59 (SE = 0.22) and a kurtosis of 1.70 (SE = 0.44).

### 9.2 Validity and reliability of the measurement data

The Cronbach’s alpha (*α* = 0.86) for the overall survey indicated good to excellent internal consistency. The composite score of the items, as measured by the Kolmogorov–Smirnov test, showed no significant deviation from a normal distribution [*D*(122) = 0.065, *p* = 0.20], and the use of Pearson correlation seemed thus warranted.

### 9.3 Potential confounders

There was no relationship between proactive or reactive cognitive control and child development, the latter measured by the HOME-21. Therefore, child development was discarded as a confounder.

### 9.4 Clinical-health psychology theory of cognitive control (Model 1–4)

#### 9.4.1 Testing the mediational models

Ten mediation analyses were conducted with the overall sample (Table 4). Reverse causal effect models displayed much lower effect sizes than the proposed models and were thus deemed unlikely to be a solution. The results, including the confidence intervals and effect sizes for each model, are depicted in Table 2. Nine out of the ten mediation models were highly significant, with large effect sizes. All except one model were thus supported. The results of the mediational models are illustrated in Table 4.

**Table 4:** Confidence intervals and effect sizes for the ten mediational models.

Model	Independent variable	Mediator variable	Dependent variable	Confidence intervals (CI)	*R^2^*
1	Proactive & Reactive Cognitive Control	Distress Tolerance	Mental Illness	(*p < 0*.001**[−0.018, −0.002])	0.37
2	Proactive & Reactive Cognitive Control	Distress Tolerance	Mental Health	(*p < 0*.001** [0.040, 0.383])	0.25
3	Proactive & Reactive Cognitive Control	Emotional Regulation	Mental Health	(*p < 0*.001** [0.153, 0.628])	0.31
4	Proactive & Reactive Cognitive Control	Emotional Regulation	Mental Illness	(*p < 0*.001** [−0.023, −0.005])	0.29
5	Proactive & Reactive Cognitive Control	Sensory Processing Problems	Mental Health	(*p < 0*.001** [0.18, 0.54])	0.28
6	Proactive & Reactive Cognitive Control	Sensory Processing Problems	Mental Illness	(*p < 0*.001** [−0.018, −0.004])	0.15
7	Negative Affect	Proactive & Reactive Cognitive Control	Mental Health	(*p < 0*.001** [−0.256, −0.012])	0.32
8	Negative Affect	Proactive & Reactive Cognitive Control	Mental Illness	CI includes zero	Ns
9	Self-Efficacy	Proactive & Reactive Cognitive Control	Mental Health	(*p < 0*.001** [−0.010, 0.401])	0.38
10	Self-Efficacy	Proactive & Reactive Cognitive Control	Mental Illness	(*p < 0*.001** [−0.314, −0.013])	0.25

*p < 0.05, **p < 0.001. Ns, not significant. All models except of model 8 are highly significant.

**Figure 2:**
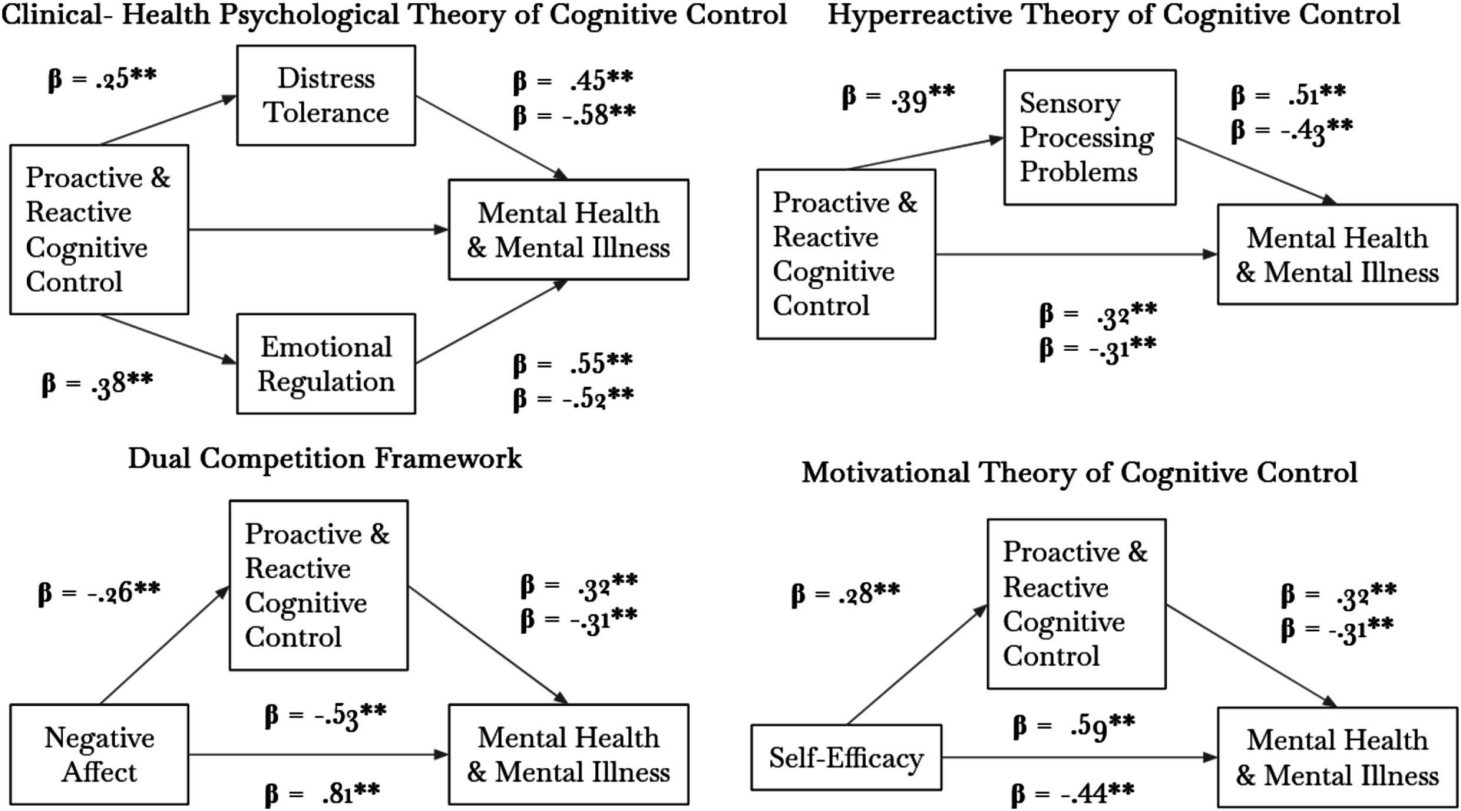
Results of the ten Preacher and Hayes mediational analyses.

The relationships between proactive and reactive cognitive control, mental health and mental illness were mediated by DT and ER. As Figure 2 illustrates, the standardized regression coefficient between all variables was highly significant. The bootstrapped standardized indirect effect did not include zero in the proposed models and was thus deemed significant. All effect sizes, as measured by eta squared, were strong.

#### 9.4.2 Hyperreactive theory of cognitive control (Model 5–6)

The relationships between proactive and reactive cognitive control, mental health and mental illness were mediated by sensory processing problems. As Figure 2 illustrates, the standardized regression coefficients between all variables were highly significant. The bootstrapped standardized indirect effect did not include zero in the proposed models and was thus deemed significant. All effect sizes, as measured by eta squared, were strong.

#### 9.4.3 Dual competition framework (Model 7–8)

The relationship between negative affect and mental health was mediated by proactive and reactive cognitive control but not by the relationship between negative affect and mental illness. As Figure 2 illustrates, the standardized regression coefficients between all variables were highly significant. The bootstrapped standardized indirect effect did not include zero for mental health-dependent variables but included zero for mental illness. Therefore, negative affect and mental health, but not mental illness, were mediated by proactive and reactive cognitive control.

#### 9.4.4 Motivation theory of cognitive control (Model 9–10)

The relationships between self-efficacy, mental health and mental illness were mediated by proactive and reactive cognitive control. As Figure 2 illustrates, the standardized regression coefficients between all variables were highly significant. The bootstrapped standardized indirect effect did not include zero in any of the proposed models and was thus deemed significant. All effect sizes, as measured by eta squared, were strong.

## 10 Discussion

The purpose of the current research was to identify and investigate leading models of the relationships among cognitive control, mental health and psychological disorders worldwide. The Norwegian model of cognitive control emphasizes that the inability to disengage from irrelevant stimuli is related to a hyperactive state of mind, which is associated with high levels of anxiety and deteriorated mental health. Motivational accounts of cognitive control highlight the decisive influence of perceived self-efficacy, which is positively related to mental health and negatively related to psychological disorders. However, clinical-health psychological theories of cognitive control focus on the influence of cognitive control on the ability to regulate emotions. The dual competition framework highlights the predominant role of negative affect.

This study rests on the assumption that reactive, but not proactive, cognitive control is related to a reduced flexibility of cognitive control (Yang and Pourtois, 2022). The dual competition framework postulates that cognitive control is primarily governed by stimuli with emotional valance (Straub et al., 2020) and that cognitive control operates via two distinct modes: proactive and reactive cognitive control (Braver, 2012). Yang and Pourtois (2022) suggested that cognitive control depends on the subtle balance between reactive and proactive control and is therefore flexible. The literature review conducted in this article identified the most predominant models on the relation between cognitive control, mental health and psychological disorders. The mediational analyses conducted in this study supported almost all the models. The models are discussed in the following.

### 10.1 Hyperreactive theory of cognitive control

The hyperractive theory of cognitive control was primarily advanced by Olsen et al. (2015, 2017, 2018). This view proposes that symptoms of anxiety, depression and other psychological disorders are mainly the result of an individual’s inability to disengage from irrelevant stimuli, implying that those individuals may not be able to “filter” relevant from irrelevant stimuli and that this results mainly from a breakdown of proactive cognitive control. These results are similar to the findings of the Parieto-Frontal Integration Theory (P-FIT) by Jung and Haier (2007), which highlights the critical role of white matter organization. The P-FIT was deemed the “best available answer to the question of where in the brain intelligence resides” (Deary et al., 2010, p. 7). Olsen et al. (2018) investigated the cognitive control strategies of children with a very low birth weight and found that those children exceedingly used a reactive mode of control. This group of participants demonstrated disrupted white matter organization, especially in the dorsolateral prefrontal cortex. In line with Olsen et al. (2015, 2017, 2018), this study established a mediational effect of sensory processing problems on the relationship between reactive cognitive control and psychological disorders and between proactive cognitive control and mental health. The observed effect sizes were only small to medium.

### 10.2 Clinical-health psychology theory of cognitive control

The Clinical-Health Psychology Theory of Cognitive Control proposes that deficits in proactive cognitive control represent a robust link to underlying neuropathophysiology because the same brain regions responsible for high DT and ER are implicated in cognitive control functioning (Aron et al., 2003; Boes et al., 2008; Daughters et al., 2017; Japee et al., 2015; Menon and Uddin, 2010; Niendam et al., 2012; Shackman et al., 2011). This view has been supported and advanced by an array of studies (Lesh et al., 2011). Further support for this model is the finding that low DT and ER strategies lead to counterproductive coping mechanisms (e.g., behavioral avoidance or suppression), which are involved in the development of psychological disorders (Jeffries et al., 2016; Larrazabal et al., 2022; Leyro et al., 2010), such as borderline personality disorder, self-injurious behavior, gambling and substance use disorder (Alker, 2024; Anestis et al., 2007; Buckner et al., 2007; Daughters et al., 2005; Daughters et al., 2008; Gratz et al., 2006; Nock and Mendes, 2008). For example, those who are consistently unable to withstand or tolerate anxiety or situations that induce negative affect may not become habituated to fear sensations and thus miss opportunities to develop strategies and self-efficacy to cope with those situations (Alker, 2024). Thus, counterproductive coping strategies not only maintain but also potentiate psychological problems. The mediation models conducted in this study support this view and yielded overall the strongest observed effect sizes.

### 10.3 Dual-competition framework

Similar to the clinical-health psychology theory of cognition, the dual competition framework proposes that negative affect influences processing efficiency. Valadez et al. (2021) reported that the compensatory process to spare performance, that is, the excessive use of reactive rather than proactive cognitive control, leads to behavioral inhibition, which in turn increases the predisposition toward psychological disorders (Hirshfeld-Becker et al., 2008). This study can only partially support this view, as proactive and reactive cognitive control did not mediate the relationship between negative affect and mental disorders. This study revealed a strong positive association between low negative affect and mental health. One reason for the lack of a mediational effect might thus be that more frequent use of reactive cognitive control itself may lead to low ER and DT by favoring counterproductive coping strategies instead of facilitating situations in the face of negative affect. This reasoning indicates that cognitive control itself leads to negative affect and not vice versa.

### 10.4 Motivation theory of cognitive control

According to motivational accounts, individuals integrate the self-efficacy of task performance and the expected reward to determine the expected value of control and subsequently adjust their allocated control (Frömer et al., 2021). Decisively, the benefits are a function of both the expected outcomes for reaching one’s aim and the likelihood of reaching that aim with a certain investment of control. In this view, impaired motivation is a central feature of mental illness, disturbed functioning, and poor mental health (Culbreth et al., 2018; Grahek et al., 2019). Low motivation and perceived self-efficacy presumably decrease the use of proactive cognitive control in favor of increasing reactive cognitive control in the face of hopelessness, which is the key characteristic of depression and an array of psychological disorders (Gan et al., 2022; Maier and Seligman, 2016; Seligman, 1972). In line with this reasoning, this study revealed a strong association between self-efficacy, proactive cognitive control and mental health and a strong relationship between lower levels of self-efficacy and the use of reactive cognitive control and the development of psychological disorders. The observed effect sizes were large in magnitude. Additionally, effect sizes were much larger for the proposed models than for the reversed causal effect models, implying that self-efficacy precedes the usage of the respective mode of cognitive control, rather than vice versa. Although causation cannot be established from a descriptive design, this finding emphasizes the paramount role of self-efficacy in how much effort might be employed in cognitive control and, therefore, which of them might be preferably and habitually employed by an individual. The findings thus shed light on the etiology of psychological disorders and the promotion of mental health by bridging the gap between motivational and clinical psychological accounts of cognitive control. Proactive cognitive control presumably relies on more effort and thus results in a more productive way to cope with negative affect in the immediate face of distress.

### 10.5 Theoretical framework of cognitive control and mental health

**Figure 3:**
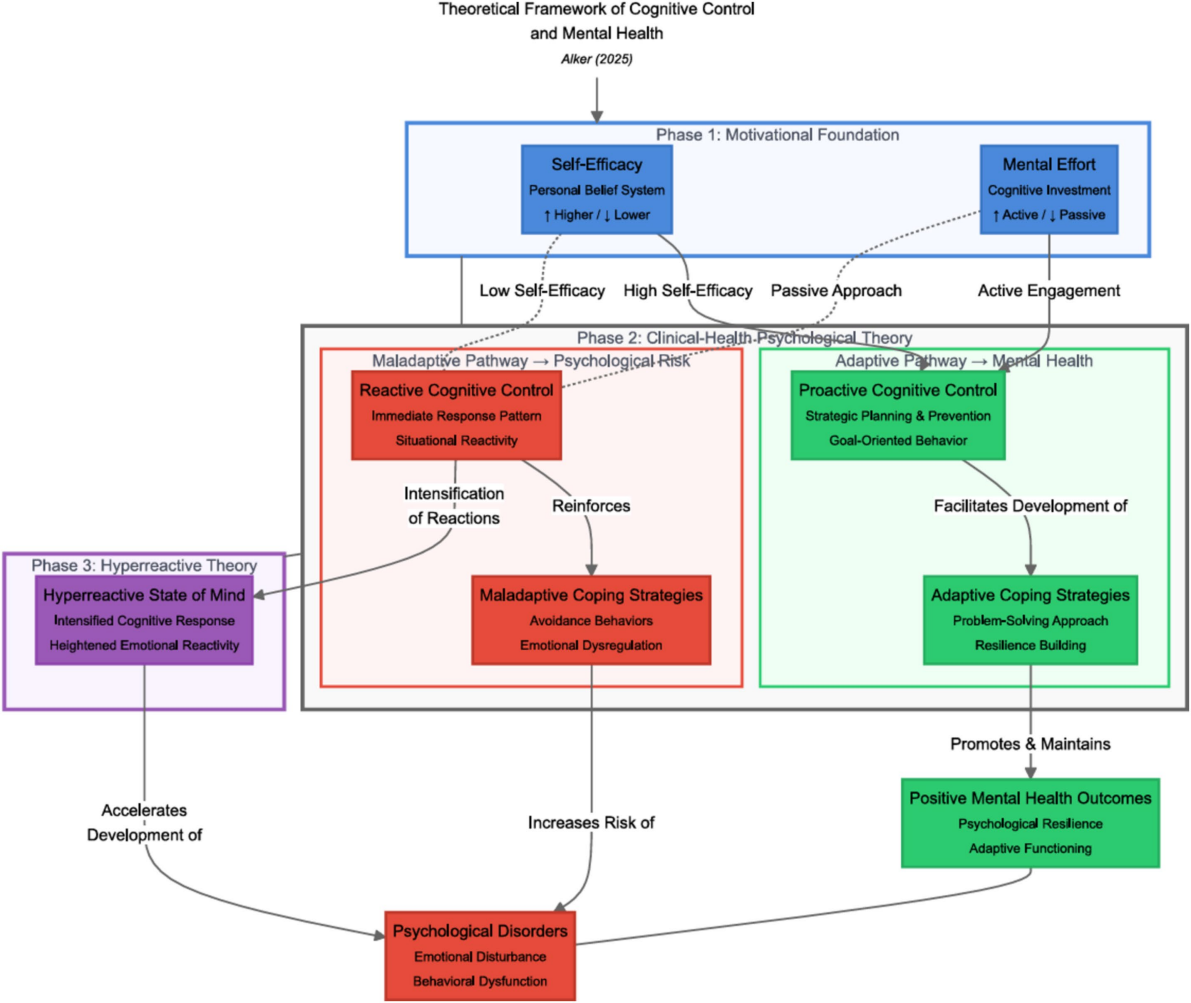
Theoretical framework of cognitive control and mental health. This framework visually represents key theories related to cognitive control. Motivational theories are highlighted in blue, emphasizing their role in fostering positive cognitive strategies. Clinical health psychological theories are marked in green, illustrating their relevance to understanding mental health and well-being. The impact on mental health and the development of psychological disorders is indicated in red, drawing attention to the potential risks associated with cognitive control strategies. Dashed lines represent the interrelatedness between the different models. The Norwegian perspective on cognitive control is highlighted in purple. Additionally, the effect sizes from reversed causal effect models—where *m* is substituted with *x* to explore the most likely causal directions—provide further support for the proposed framework.

Based on the findings of this study, a model can be proposed taking into account the models with the largest effect sizes (Figure 3). This model can be divided into antecedents (the motivational account of cognitive control) and descendants (sequelae of cognitive control) that follow the respective mode of functioning, that is, proactive versus reactive cognitive control, which are in an intermediate position. By using this model, consequences of frequent use of the respective mode of cognitive control are illustrated within the specific model. This model is subject to further scientific studies.

High self-efficacy and motivation, which are needed to engage in positive control and are positively associated with mental health, are marked in blue. Low self-efficacy or motivation putatively decreases the effort needed for the use of positive cognitive control; thus, compensatory processes, that is, reactive cognitive control, intervene to spare the performance of a breakdown of proactive cognitive control. Frequent use of reactive cognitive control, in turn, is positively related to the development of psychological disorders and negatively related to mental health.

The frequent use of proactive cognitive control increases the tendency to use adaptive strategies in the face of internal and external distressing events, which supposedly improves mental health and reduces vulnerability to psychological disorders. In contrast, frequent use of a reactive mode of cognitive control hinders the individual from “filtering” or disengaging from irrelevant stimuli, thus predisposing them to psychological disorders. This umbrella model of cognitive control thus combines both motivational and emotional theories of cognitive control.

### 10.6 Limitations

This study employed a descriptive design. Although causal effect models are automatically calculated by Preacher and Hayes (2014) bootstrapped mediational analyses, no causations can be established from a descriptive design (Grimes and Schulz, 2016). Furthermore, this study relies on the assumption that flexibility is the underlying hallmark of proactive versus reactive cognitive control (Mäki-Marttunen et al., 2019; Yang and Pourtois, 2022). However, both modes of control are related but distinct from each other (Braver, 2012). Although the use of cognitive flexibility can be considered the paramount characteristic of the respective modes, this can merely be deemed an indirect and incomplete capture of proactive and reactive cognitive control. This is, however, currently the only available method to compare the leading models of cognitive control. Experimental studies are recommended to establish a causal link between motivational factors, cognitive control, mental health and psychological disorders. The reliance on self-reported Likert-scale measures to assess cognitive control presents challenges, as such tools are prone to biases and may not fully capture cognitive constructs. Self-reported measures can be influenced by social desirability bias, recall bias, and individual differences in self-perception.

### 10.7 Strengths

This is the first study to identify and compare the leading models of the relationships among cognitive control, the etiology of psychological disorders and the promotion of mental health. Furthermore, this study employed mediational analyses developed by Hayes and Preacher (2014) to compare the leading models of cognitive control and the impact of cognitive control on mental health and the exhibition of psychological symptoms. The Preacher and Hayes (2014) approach does not require a normal distribution of the sample population since it uses a bootstrapping method, nor does it require significant coefficients *a* and *b* to confirm a mediational effect of *m* on the relation between *x* and *y*. The bootstrapping approach is a viable method to circumvent the statistical problems usually exhibited by other mediational analyses. Finally, this study is the first to investigate both mental illness and mental health as two separate but related continua and the relationship to cognitive control. Teng et al. (2015) argue that an exclusive focus on the identification of mental illness or mental health runs the risk of missing data for individuals who exhibit low or high levels of the other mental illness or mental health.

### 10.8 Future research

To address the limitations, future research could incorporate objective measures of cognitive control, such as neuroimaging techniques, behavioral tasks, or physiological assessments. These methods could provide a more comprehensive and accurate understanding of cognitive control processes. The study’s findings are based on a sample composed exclusively of university students and clinical staff. This homogeneity limits the generalizability of the results, as some demographic and cultural variations are not included. To enhance the validity of the findings, future studies are recommended to include a diverse sample that represents different age groups, educational levels, and cultural backgrounds. Greater diversity in the sample will increase the external validity.

Future research may elucidate the intricate and complex relationship between proactive and reactive modes of control and the development of psychological disorders and the promotion of mental health by establishing causal relationships for the proposed direction. Therapeutic approaches may also integrate motivational aspects to improve therapeutic outcomes. Clarifying whether cognitive control precedes negative emotional states and affect would pay attention to the paramount role of cognitive control and cognition in the etiology of psychological disorders. The impact of positive emotions on pro-and reactive cognitive control was not investigated in this study. Although this study revealed no direct mediating effect of negative affect on the relationship between cognitive control and mental disorders, there might nonetheless be an effect of positive emotions on levels of perceived self-efficacy, which increases the likelihood of exhibiting the high effort needed for proactive enactment cognitive control. Self-efficacy, in turn, can explain why individuals with the same skills may adjust and adapt poorly, adequately, or exceptionally to life’s infinite challenges. Although causations cannot be established by a correlational design, lower effect sizes of the reversed causal effect models support the proposed directions and the leading models of cognitive control. Further experiments are recommended to support the Theoretical framework of cognitive control and mental health.

The inflammatory hypothesis suggests that chronic inflammation may contribute to cognitive dysfunction and mental health disorders. Elevated levels of pro-inflammatory cytokines, such as interleukin-6 (IL-6) and tumor necrosis factor-alpha (TNF-*α*), have been associated with impaired cognitive control and increased risk of depression and anxiety. Exploring these biochemical mechanisms could offer valuable insights into the complex interplay between cognitive control, mental health, and psychological disorders. Furthermore, incorporating biochemical perspectives could help identify potential biomarkers for cognitive control deficits and mental health disorders, paving the way for more targeted and effective therapeutic interventions. By integrating neuroanatomical, biochemical, and psychological perspectives, future research can provide a more holistic understanding of the factors influencing cognitive control and mental health.

## 11 Conclusion

This study identified and investigated four leading models of the relationships among cognitive control, mental health and psychological disorders. The findings of this study highlight the pivotal role of cognitive control or executive attention in the etiology of psychological disorders and mental health by illuminating its role in ER strategies. This research strongly supports the notion that the same brain regions responsible for ER strategies are responsible for intact proactive cognitive control and the development of resilient mental health. Cultivating a proactive mode of cognitive control not only fosters ER strategies to cope with negative emotions in the face of distress and future stressors but can also be unequivocally identified as a paramount feature in ameliorating or altering a person’s response to hazardful events that predispose a maladaptive outcome in the face of uncertainty, distress and mental health problems.

## Data Availability

The raw data supporting the conclusions of this article will be made available by the authors, without undue reservation.
