# Correction: Cognitive control: modeling the impact on mental health

**DOI:** 10.3389/fpsyg.2026.1852604

**Published:** 2026-04-21

**Authors:** Leon Alker

**Affiliations:** Department of Psychology, Philipps University Marburg, Marburg, Germany

**Keywords:** cognitive control, mental health, affective neuroscience, computational modeling, clinical neuropsychology, mind control

In the published article, the acronyms SONA and SoSci were incorrectly defined in Section 8.1 (Participants).

The following correction has been made:

“This study was carried out in accordance with relevant guidelines and regulations and was approved by the ethics committee of Philipps-University Marburg, Germany. Informed consent was obtained from all 122 participants. Ninety-five participants were students, 27 of whom were employees of a university hospital. Students were recruited via the SONA first-year practicum and were then forwarded to the online survey platform SoSci Survey for study participation. SONA is an online participant management system used to recruit and schedule student participants, typically in exchange for course credit. SoSci Survey is an online survey platform used to administer questionnaires and collect data.”

The original version of this article has been updated.

